# Robust Copper Metal–Organic Framework-Embedded Polysiloxanes for Biomedical Applications: Its Antibacterial Effects on MRSA and In Vitro Cytotoxicity

**DOI:** 10.3390/nano11030719

**Published:** 2021-03-12

**Authors:** Kihak Gwon, Youngmee Kim, Hyunjun Cho, Seonhwa Lee, So-Hyeon Yang, Sung-Jin Kim, Do Nam Lee

**Affiliations:** 1 Ingenium College of Liberal Arts (Chemistry), Kwangwoon University, Seoul 01897, Korea; khgwon@kw.ac.kr (K.G.); seonhwalee@kw.ac.kr (S.L.); 2 Department of Chemistry and Nano Science, Institute of Nano-Bio Technology, Ewha Womans University, Seoul 03760, Korea; ymeekim@ewha.ac.kr (Y.K.); auung22@ewhain.net (S.-H.Y.); sjkim@ewha.ac.kr (S.-J.K.); 3 Department of Chemistry, Dongguk University, Seoul 04620, Korea; vchol1212@dgu.ac.kr

**Keywords:** Cu-MOF, polysiloxane (PS), hydrosilylation, antibacterial agent, cytocompatibility, biomedical application

## Abstract

Polysiloxanes (PSs) have been widely utilized in the industry as lubricants, varnishes, paints, release agents, adhesives, and insulators. In addition, their applications have been expanded to include the development of new biomedical materials. To modify PS for application in therapeutic purposes, a flexible antibacterial Cu-MOF (metal–organic framework) consisting of glutarate and 1,2-bis(4-pyridyl)ethane ligands was embedded in PS via a hydrosilylation reaction of vinyl-terminated and H-terminated PSs at 25 °C. The bactericidal activities of the resulting Cu-MOF-embedded PS (PS@Cu-MOF) and the control polymer (PS) were tested against *Escherichia coli*, *Staphylococcus aureus*, and methicillin-resistant *Staphylococcus aureus*. PS@Cu-MOF exhibited more than 80% bactericidal activity toward the tested bacteria at a concentration of 100 μg⋅mL^−1^ and exhibited a negligible cytotoxicity toward mouse embryonic fibroblasts at the same concentration. Release tests of the Cu(II) ion showed PS@Cu-MOF to be particularly stable in a phosphate-buffered saline solution. Furthermore, its physical and thermal properties, including the phase transition, rheological measurements, swelling ratio, and thermogravimetric profile loss, were similar to those of the control polymer. Moreover, the low cytotoxicity and bactericidal activities of PS@Cu-MOF render it a promising candidate for use in medicinal applications, such as in implants, skin-disease treatment, wound healing, and drug delivery.

## 1. Introduction

The use of antimicrobial plastics has attracted considerable attention in medicinal and biomedical engineering fields in recent years. Medical polymers must be harmless to the living body, wherein they should exhibit a high biocompatibility with tissues, cells, and blood to avoid immune rejection reactions, and they must maintain their necessary physical and mechanical properties within the host [1,2,3,4,5,6,7,8,9]. Polysiloxane (PS) materials exhibit interesting properties including good thermal and oxidative stabilities, dielectric insulating properties, fouling release properties, and excellent biocompatibilities [10,11]. As a result, PSs have been widely utilized in the industry as lubricants, varnishes, paints, release agents, adhesives, and insulators [12,13,14,15,16,17,18]. In addition, due to their physicochemical properties, the uses of PS have been expanded to include medical materials such as tubes, pre-coating needles, syringes, artificial heart valves, pacemakers, and contact lenses [19,20,21]. Many studies have therefore been conducted to develop a surface modification method to impart antibacterial properties to PS for its use in biological applications. In fact, many literature reports have indicated that surface-modified PSs exhibit a good antibacterial effect when coated with silver nanoparticles, organic antibiotics, quaternary ammonium salts, chitosan, and antimicrobial drugs or other materials [22,23,24,25,26,27,28,29,30,31,32,33].

Recently, metal–organic frameworks (MOFs), which are porous coordination materials composed of organic ligands and metal ions, have received considerable interest as antibacterial materials for biological, environmental, and food applications, owing to their sustained release capability, porosity, structural flexibility, and antibacterial properties [34,35,36,37,38]. However, the release of excess metal ions from MOFs can have toxic effects on the host tissues, as well as microbes, and so it is desirable to improve their stabilities [39,40,41]. Considerable efforts have therefore been expended to improve the antibacterial durabilities of such materials and to reduce any adverse effects. This has been achieved by covalently introducing biocidal functions to the polymer [42,43]. Recently, our group developed a series of stable antibacterial MOFs (i.e., Cu-, Co-, and Zn-based MOFs), which were embedded into photo cross-linkable hydrogels for drug delivery. Importantly, the Cu-MOF-embedded hydrogels exhibited an excellent bactericidal activity against both *Escherichia coli* and *Staphylococcus aureus*, with no observed cytotoxicity [44]. Notably, we reported that Cu-MOFs containing glutarate (Glu) and bis(4-pyridyl) ligands showed very excellent bioactivity toward various bacteria [45].

Thus, we herein report the development of a simple antibacterial Cu-MOF-embedded PS scaffold with negligible toxicity, which is prepared via a hydrosilylation method. More specifically, an antibacterial Cu-MOF containing Glu and 1,2-bis(4-pyridyl)ethane (bpa) is prepared, using a previously reported method, and embedded on PS via hydrosilylation reactions with vinyl-terminated PS (PS-vinyl) and hydrogen-terminated PS (PS-H) at 25 °C. Their characterizations were investigated using powder X-ray diffraction (PXRD), differential scanning calorimetry (DSC), thermogravimetric analysis (TGA), Fourier-transform infrared spectroscopy (FT-IR), rheological measurements, scanning electron microscopy–energy dispersive X-ray spectroscopy (SEM-EDS), and inductively coupled plasma mass spectrometry (ICP-MS). Overall, this study focuses on the development of a new type of antibacterial PS, which can be widely applied in drug delivery, wound healing, and antibacterial coatings for medical devices and implants, as determined by the evaluation of the antibacterial activity against *Escherichia coli (E. coli), Staphylococcus aureus (S. aureus)*, and methicillin-resistant *Staphylococcus aureus* (MRSA). The in vitro cytotoxicity of PS and PS@Cu-MOF against mouse embryonic fibroblasts is also examined and discussed.

## 2. Materials and Methods

### 2.1. Preparation of the Cu-MOF

The Cu-MOF, consisting of glutarate (Glu) and bpa ligands, was synthesized hydrothermally via a previously reported method, and its formula was determined to be (Cu_2_ (Glu)_2_(bpa))·3H_2_O [45,46]. More specifically, a mixture of Cu(NO_3_)_2_·3H_2_O (2.0 mmol, 99%, Sigma-Aldrich, St. Louis, MO, USA), glutaric acid (2.0 mmol, 99%, Sigma-Aldrich, St. Louis, MO, USA), and bpa (1.0 mmol, 99%, Sigma-Aldrich, St. Louis, MO, USA) was prepared in distilled water containing 1.0 M NaOH, and the hydrothermal reaction was carried out at 80 °C as reported. The obtained Cu-MOF was analyzed by PXRD and FT-IR.

### 2.2. Preparation of PS and the Cu-MOF-Embedded PS (PS@Cu-MOF)

The Cu-MOF-embedded PS (PS@Cu-MOF) was prepared through a hydrosilylation reaction between two PS components with a TiO_2_ additive in the presence of a Pt catalyst (KE-1300 and CAT-1300, Shin-Etsu Chem. Co., Tokyo, Japan) and Cu-MOF. More specifically, the vinyl-terminated PS (PS-vinyl) containing the Pt catalyst and TiO_2_ additive was mixed thoroughly with the Cu-MOF (100 μg⋅mL^−1^) for 10 min, and then the hydrogen-terminated PS (PS-H) was added to the mixture (10:1 weight ratio of vinyl/hydrogen) and thoroughly mixed for an additional 5 min. Subsequently, the mixture was degassed under vacuum and pipetted into a 50 × 50 mm^2^ glass mold prior to vulcanizing for 24 h at 25 °C. PS alone (i.e., without the Cu-MOF) was also prepared in the same manner and employed as a positive control. The obtained polymers were characterized by PXRD, DSC, TGA, SEM-EDS, Rheometer, and ICP-MS.

### 2.3. Instrumentation

PXRD patterns of Cu-MOF, PS, and PS@Cu-MOF were recorded using a Rigaku MiniFlex diffractometer (Rigaku Corp, Neu-Isenburg, Germany). The FT-IR spectrum was measured on a Bio-Rad FTS 135 spectrometer (Hercules, CA, USA). DSC runs were carried out using a DSC 214 Polyma (NETZSCH, Burlington, MA, USA). TGA was performed using a TG 209 F3 Tarsus^®^ instrument (NETZSCH, Burlington, MA, USA). The surface morphology and elemental composition of PS and the PS@Cu-MOF scaffolds were characterized using SEM-EDS (FE-SEM, JEOL JSM-5800F, Peabody, MA, USA). The mechanical properties of PS and PS@Cu-MOF were evaluated using a TA rheometer (Discovery HR 10, New Castle, DE, USA). The degradation of PS@Cu-MOF was carried out by ICP-MS (Agilent Marker 7700, RF Generator Power 1550 W, Tokyo, Japan). The colorimetric absorbance was determined using a microplate reader (Synergy H1, BioTek, Winooski, VT, USA), and the live/dead double-stained cells were imaged by fluorescence microscopy (IX83, Olympus, Center Valley, PA, USA). 

### 2.4. Mechanical Properties of the PS@Cu-MOF

For the rheological measurements, PS@Cu-MOF and PS (8 mm in diameter, 1 mm thickness) were prepared and soaked in 0.01 M pH 7.4 PBS for 2 d at 37 °C. The frequency sweep test (from 0.01 to 100 rad/s) was tested at 1% strain and 25 °C. To quantify the swelling ratios of PS and PS@Cu-MOF, both samples were allowed to reach their fully swollen states and then weighed (Ws) after drying the outer surface carefully with a tissue. Each sample was then lyophilized for two days and weighed (Wd), and the swelling ratios were calculated as follows:

(1) $$\mathrm{Swelling}\;\mathrm{ratio}=\frac{\mathrm{Ws}}{\mathrm{Wd}}$$

### 2.5. Degradation and Metal Ion Release Tests

To assess whether PS@Cu-MOF could release its constituent metal ions, each of the four solutions containing PS@Cu-MOF was prepared at concentration of 1 mg⋅mL^−1^ in 0.9% phosphate-buffered saline (PBS) and stirred at 25 °C for 6, 12, 24, or 48 h. Subsequently, each sample was subjected to centrifugation, and the supernatant was separated from the reaction tube. The quantity of Cu(II) ions released was measured by ICP-MS analysis of the supernatant. The degree of degradation was expressed as the concentration of Cu(II) ions released into the PBS, in μg⋅mL^−1^, at each release test time.

### 2.6. Antibacterial Tests

Antibacterial activity tests on PS and PS@Cu-MOF were carried out using the following three strains of bacteria: *E. coli* (ATCC 8739), *S. aureus* (ATCC 6538P), and MRSA (ATCC 33591), according to a previously reported colony counting method [44]. More specifically, to measure the antibacterial activity of PS@Cu-MOF against the three bacterial strains, three specimens of PS@Cu-MOF and a stomacher film for the blank (5 × 5 ± 0.2 cm, within 1 cm thickness) were prepared and tested according to the following method. As a positive control, PS (without the MOF) was also prepared and evaluated. The sample was completely dried after wiping three times with an ethanol-soaked gauze. The pre-cultured test bacteria with a concentration of ~10^5^ cfu ⋅mL^−1^ (colony-forming units) were routinely inoculated. Each test sample was placed in a Petri dish with the test side up. Subsequently, an aliquot (200 μL) of the test solution was inoculated onto each test piece. The film placed on the dropped test bacterium was lightly pressed to cover it with the test bacterium. The test sample, and the control inoculated with the test strain, were incubated for 24 h at 37 °C. After inoculation, the uncoated test pieces bearing the test strain were immediately separated using sterilized tweezers, and SCDLP medium (10 mL) was added to wash off the test bacteria. The viable cell count of this washing solution was measured. Subsequently, 1 mL of the washing solution was added to the test tube containing 9 mL of physiological saline solution and mixed well. The washing solution was then diluted stepwise, and an aliquot (100 μL) of the diluted solution was placed onto nutrient agar plates and incubated for 24 h at 37 °C. All experiments were performed in triplicate.

### 2.7. Cytotoxicity Assays

The cytotoxicity of the prepared PS@Cu-MOF was then evaluated as described previously [44,47]. More specifically, a fibroblast monolayer was formed on a collagen type I coated glass slide at a density of 3 × 10^4^ cells⋅cm^−1^ and incubated for 3 h. Non-adhered cells were then rinsed with Dulbecco’s Modified Eagle Medium (DMEM supplemented with 10% FBS, 200 IU⋅mL^−1^ penicillin, and 200 μg⋅mL^−1^ streptomycin), transferred to a 12-well plate, and cultured in the cell culture medium at 37 °C in a humidified incubator containing 5% CO_2_. The following day, the prepared PS@Cu-MOF (100 μg⋅mL^−1^ Cu-MOF-embedded PS, 1.5 cm diameter) was carefully placed on the cell monolayer and incubated for either 1 or 3 d. Thereafter, the cells were stained using a live/dead double-staining protocol [48]. The stained cells were imaged by fluorescence microscopy, and the cell viability was quantified by calculating the percentage of live cells among the total cells. In addition, the cytotoxicity of the extract of PS@Cu-MOF was also evaluated by measuring the cell viability in the presence of the extract solution [49]. For this purpose, PS@Cu-MOF was incubated with the cell culture medium (3 cm^2^ mL^−1^) for 24 h to obtain the extract solution, and the cells were seeded in a 24-well plate at a density of 5 × 10^4^ cells per well. After incubating at 37 °C for 24 h, the culture medium was replaced in each well using the DMEM medium containing the extract solution (200 μL). After incubating for 24 h, the DMEM-containing extract solution was replaced with a fresh medium (200 μL). Subsequently, an aliquot (20 μL) of the MTS cell proliferation assay kit (Abcam, Cambridge, MA, USA) was added to each well, and incubation was carried out for an additional 4 h. Finally, the colorimetric absorbance of the produced formazan was measured at 490 nm using a microplate reader. The cell viability was calculated using the following equation:

(2) $$\mathrm{cell}\;\mathrm{viability}\;\left(\%\right)=\left(\frac{{\mathrm{OD}}_{\mathrm{sample}}-{\mathrm{OD}}_{\mathrm{blank}}}{{\mathrm{OD}}_{\mathrm{control}}-{\mathrm{OD}}_{\mathrm{blank}}}\right)\times 100$$

where OD_sample_ represents the absorbance of the wells containing the extract solution, OD_control_ represents the absorbance of the wells containing only the culture medium, and OD_blank_ represents the absorbance of the wells that contained no cells. The cytotoxicity of PS was also determined using the above method.

## 3. Results and Discussion

### 3.1. Preparation of the Cu-MOF-Embedded PS (PS@Cu-MOF)

**Figure 1:**
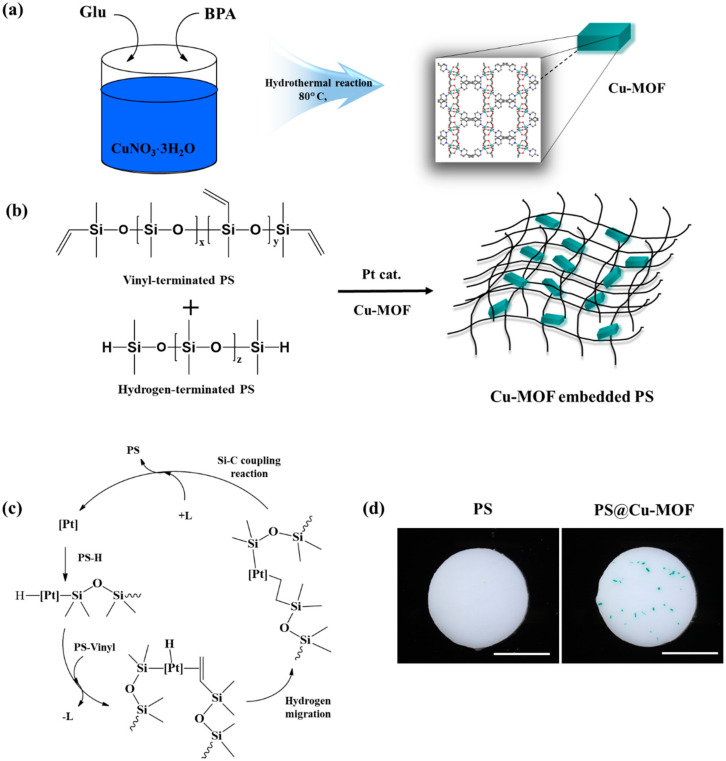
(**a**) Schematic illustration of the synthesis of Cu-MOF. (**b**) Hydrosilylation of vinyl-terminated PS and hydrogen-terminated PS with Cu-MOF in the presence of a Pt catalyst. (**c**) Curing mechanism for the hydrosilylation of PS-vinyl and PS-H containing a Pt catalyst. (**d**) Photographic images of PS and the PS@Cu-MOF. Scale bar: 0.5 cm.

As described in Figure 1a, the Cu-MOF was synthesized by a hydrothermal reaction between Glu, bpa, and CuNO_3_⋅3H_2_O [45]. Its bulk structure was confirmed by PXRD measurements and FT-IR spectroscopy. As illustrated in Figure S1, the main crystalline peaks of Cu-MOF appeared at 2θ = 6.8, 13.5, and 14.9°, which corresponded to the (200), (400), and (420) crystal planes, respectively, and are consistent with previous reported literature [46]. The Cu-MOF sample also showed peaks attributed to C-H stretching in the region between 650 and 900 cm^−1^, while the peaks at 1602 and 1412 cm^−1^ were attributed to bidentate inter cluster bridges and to the stretching bands of ν(C–O), respectively (Figure S2) [48]. It was also determined that the crystal structure of Cu-MOF contained dinuclear Cu_2_ units bridged by Glu ligands that formed 2D sheets connected by bpa ligands to give a 3D framework (Figure S3). We prepared PS@Cu-MOF via the facile hydrosilylation reaction of PS-vinyl and Cu-MOF with PS-H in the presence of a Pt catalyst and TiO_2_ additive for 24 h at 25 °C (Figure 1b). The proposed curing mechanism of PDMS was initiated by the insertion reaction of PS-H to the Pt catalyst, followed by PS-vinyl to the Pt-complex via a releasing ligand, elimination of PS by a Si-C coupling reaction, and re-coordination of the released ligand to the Pt catalyst as the final step (Figure 1c). Control PS was prepared by the same manner without the incorporation of Cu-MOF [50,51]. As shown in Figure 1d, both PS and the PS@Cu-MOF were successfully prepared as thin films, and Cu-MOF was successfully incorporated into the PS during the hydrosilylation process.

### 3.2. Characterization of the PS@Cu-MOF

**Figure 2:**
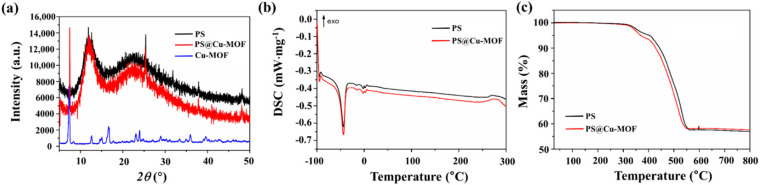
Characterization of PS and PS@Cu-MOF; (**a**) PXRD patterns, (**b**) DSC scans, and (**c**) TGA profiles.

The PXRD patterns of PS, Cu-MOF, and PS@Cu-MOF were analyzed to study the nature of crystallinity of each sample. As illustrated in Figure 2a, two broad intense peaks were observed for PS and PS@Cu-MOF, namely a large peak at ~12.8° and a smaller and broader peak at 22.6°, which were assigned to PS and TiO_2_, respectively, and corresponded with the previous reported literature [52,53]. Furthermore, PS@Cu-MOF exhibited additional crystalline peaks at 2θ = 6.8°, pertaining to the crystal planes of Cu-MOF (Figure S1). These results confirm the successful embedding of Cu-MOF in the PS networks. Figure 2b shows typical DSC scans performed at identical heating rates for the PS and PS@Cu-MOF samples. The glass transition temperatures (T_g_) of both samples were observed similarly below −100 °C, indicating that the T_g_ was not affected by the embedding of Cu-MOF on the PS. In addition, a single melting peak at around −40 °C was observed for the two PS-based species, which is common in cross-linked PS materials owing to the inability of the crosslinks to crystallize [54]. The thermogravimetric analyses of PS and PS@Cu-MOF were also carried out, wherein a single weight loss step (43.0 and 42.3%, respectively, at 511 °C) and similar thermal degradation paths were observed in an inert atmosphere (Figure 2c). This decomposition tendency suggests that the two PS networks are not significantly different from one another, i.e., the incorporation of Cu-MOF does not significantly affect the PS network. FT-IR spectral analysis was also performed. Figure S4 shows four strong and sharp peaks: the asymmetric C-H bending at 2961 cm^−1^, the symmetric C–H bending at 1258 cm^−1^, the asymmetric Si–O–Si stretching at 1009 cm^−1^, and Si-C stretching at 785 cm^−1^ in both PS@Cu-MOF and PS samples, whereas the Cu-MOF related peak did not occur from PS@Cu-MOF due to their low concentration in PS [55]. 

**Figure 3:**
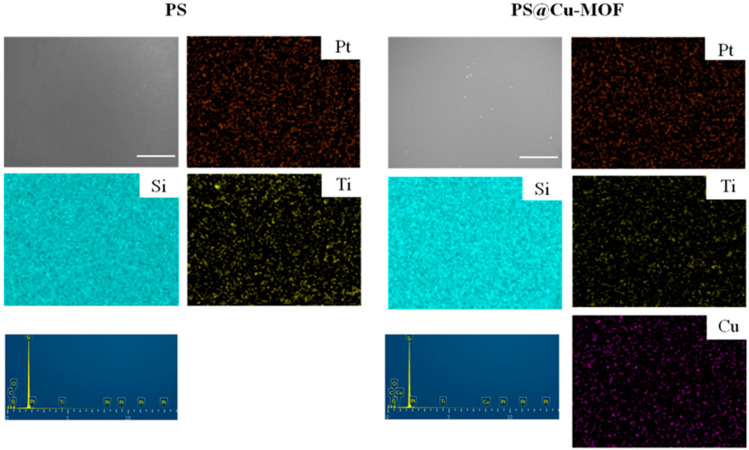
SEM images of PS and PS@Cu-MOF with the accompanying EDS spectra and corresponding elemental maps. Scale bar: 50 μm.

Moreover, analysis by SEM-EDS was carried out to obtain the morphologies and chemical compositions of the control PS and the PS@Cu-MOF. Figure 3 shows representative SEM images in addition to the corresponding mapping results for both species. More specifically, Si, Ti, and Pt were evenly spread throughout both networks (C and O were skipped), and the additional element of Cu was also found in the EDS spectrum of PS@Cu-MOF. These data confirm the embedding of Cu-MOF on PS following the above-described hydrosilylation reaction and agree with aforementioned PXRD patterns. 

### 3.3. Mechanical Properties of PS@Cu-MOF

**Figure 4:**
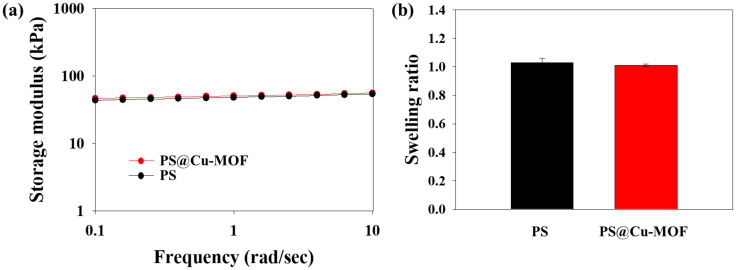
Physical properties of PS and PS@Cu-MOF. (**a**) Storage modulus and (**b**) swelling ratio of PS and PS@Cu-MOF (n = 4).

The storage moduli of PS and PS@Cu-MOF were measured to analyze their mechanical properties. As shown in Figure 4a, PS@Cu-MOF exhibited stable rheological properties (50.6 ± 0.2 kPa) and a similar modulus to PS (49.8 ± 0.3 kPa). Figure 4b shows the swelling behaviors of PS@Cu-MOF and PS, where low swelling ratios (1.01 ± 0.01 and 1.03 ± 0.03) are apparently due to the inherent hydrophobicity of PS. These results therefore indicate that PS@Cu-MOF has a polymeric network structure very similar to that of the control PS, and that the small amount of Cu-MOFs does not significantly affect the mechanical strength.

### 3.4. Antibacterial Activity Tests

**Figure 5:**
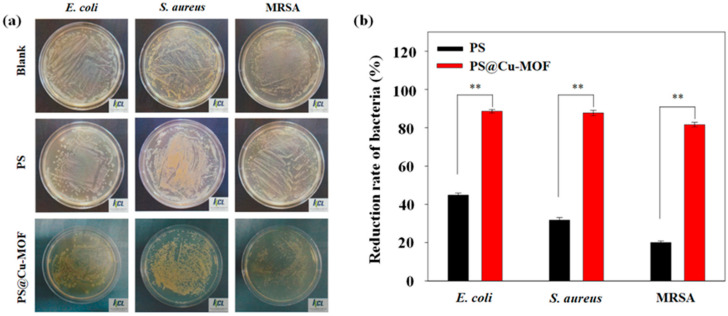
(**a**) Representative images of bacteria grown on PS and PS@Cu-MOF after incubation for 24 h: Top (blank), middle (PS), and bottom (PS@Cu-MOF); left to right: *E. coli*, *S. aureus*, and MRSA. (**b**) The antibacterial efficiency of PS and PS@Cu-MOF towards *E. coli*, *S. aureus*, and MRSA (means ±standard deviation with n = 3; **: *p* < 0.01).

The antibacterial activity of Cu-MOF has been previously reported against various strains of bacteria, including Gram-negative and Gram-positive bacteria, with Cu-MOF exhibiting excellent bactericidal properties [45]. We therefore decided to embed an antibacterial Cu-MOF to the PS to develop a new medicinal sheet, and we tested the bactericidal effects of both PS and the prepared PS@Cu-MOF against *E. coli*, *S. aureus*, and MRSA at 100 μg⋅mL^−1^ to evaluate the material potential for medicinal application. As shown in Figure 5 and Table 1, the bactericidal effect of the control PS increased in the following order: MRSA (20.0%) < *S. aureus* (31.8%) < *E. coli* (44.8%); this can be attributed to the antibacterial properties of the Pt catalyst and the TiO_2_ additive [56,57]. Upon embedding the Cu-MOFs in PS, the bactericidal effect was significantly increased as follows: MRSA (81.6%) < *S. aureus* (87.7%) < *E. coli* (88.8%). These results demonstrate that PS@Cu-MOF exhibits an excellent bactericidal effect on both Gram-negative bacteria (*E. coli*) and Gram-positive bacteria (*S. aureus*, MRSA) and shows better antibacterial activity than previously reported antibacterial polysiloxanes [58]. Graphene oxide-catechol composite (GO-DMA) was modified onto the surface of PDMS. The antibacterial activity of GO-DMA-PDMS was evaluated against *E. coli* and *S. aureus*. Antibacterial GO-DMA-PDMS killed *S. aureus* (42%) and *E. coli* (37%). Based on this result, Cu-MOF embedded PS is more bioactive than the previously published antibacterial surface-coated PDMS. 

### 3.5. Ion Release Tests

**Figure 6:**
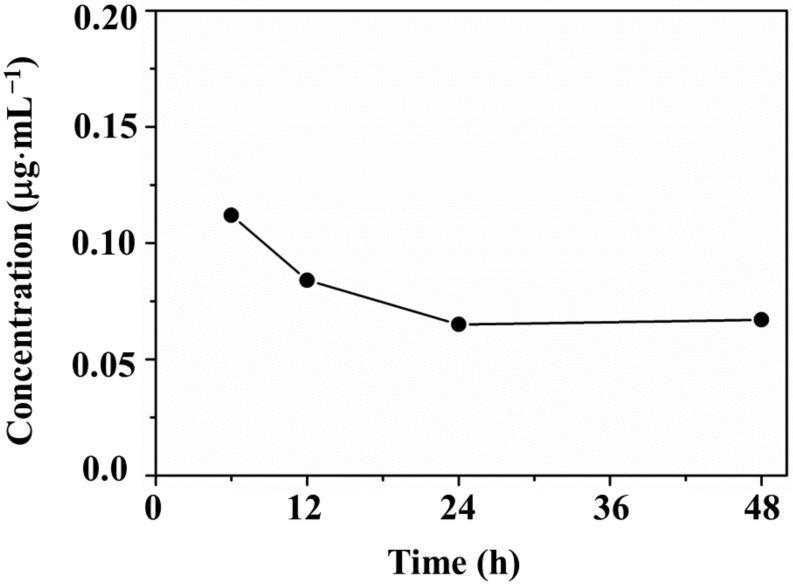
Concentration of Cu(II) ions released from PS@Cu-MOF (1 mg) in 0.9% PBS (1 mL).

Degradation tests were then performed on the PS@Cu-MOF in 0.9% PBS at 25 °C for 6, 12, 24, and 48 h, and the quantity of copper metal ions released from Cu-MOF was measured by ICP-MS. As shown in Figure 6, the concentration of metal ions released from PS@Cu-MOF increased to 0.112 μg⋅mL^−1^ during the initial 6 h, and then decreased until the 24 h point (0.065 μg⋅mL^−1^), after which it slightly increased again up to 48 h (0.067 μg⋅mL^−1^). The average concentration of Cu(II) ions released from PS@Cu-MOF after 24 h, i.e., 0.089 μg⋅mL^−1^, was lower than in the case of the Cu-MOF alone [37,38]. These results indicate that PS@Cu-MOF maintains its robust framework on the polymer network while also exhibiting excellent bactericidal properties against the various bacterial strains without the participation of released metal ions.

### 3.6. Cytotoxicity of PS@Cu-MOF

**Figure 7:**
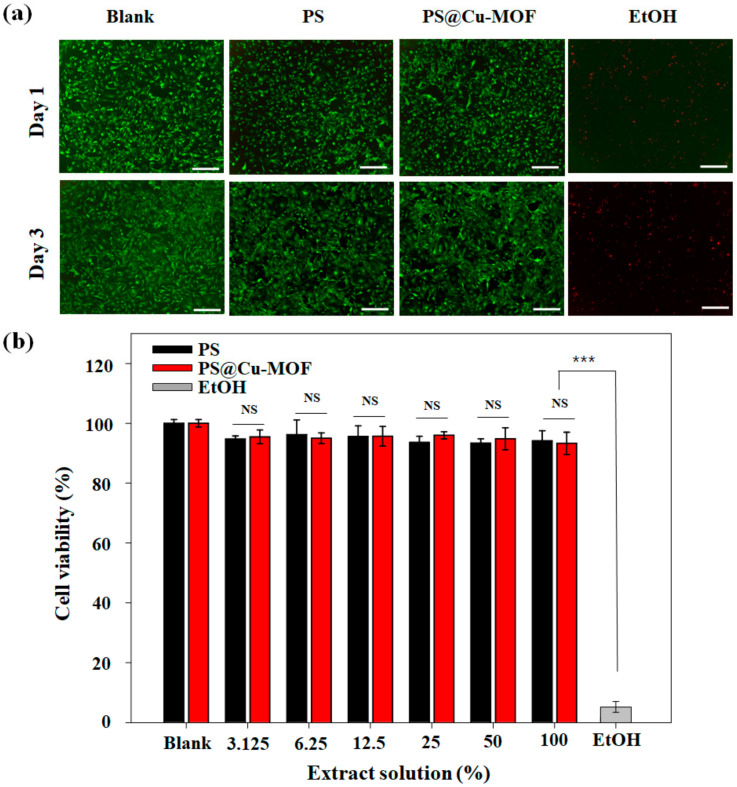
(**a**) Staining images of the live/dead MEFs after contact with PS only or PS@Cu-MOF for 1 or 3 d. Cells cultured without any PS contact were used as a positive control. As a negative control, cells exposed to EtOH were used. (**b**) In vitro cytotoxicity of the extract solution of PS and PS@Cu-MOF to MEFs (n = 4); means ± standard deviation with n = 4; NS: not significant; *** *p* < 0.001. Scale bar: 200 μm.

Finally, we studied the cell biocompatibility of PS@Cu-MOF based on a direct contact method [47]. To verify the toxicity of the PS itself, PS (i.e., without the Cu-MOF) was prepared using the same method employed for its use as a positive control. Furthermore, as an additional positive control (blank), a mouse embryonic fibroblast (MEF) monolayer without any PS or PS@MOF contact was prepared. As a negative control, an MEF monolayer contact with a 10% EtOH solution was used. The viability of the MEF monolayer was monitored using live/dead staining as well as an MTS assay. As shown in the fluorescence microscopy images (Figure 7a), the cell viabilities for the blank, PS, and PS@Cu-MOF (100 μg⋅mL^−1^) samples exceeded 98% for 1 d after culture, whereas in the case of the MEF monolayer, the majority of cells died upon exposure to EtOH. It was found that the cells gradually adhered, spread, and flattened on the surface after seeding, prior to forming a confluent-like layer that survived for 3 d; this was not observed in the case of the EtOH group. These results indicate that neither the MOF-embedded PS nor the PS alone were toxic to the cells. Further quantification was carried out using an MTS assay (Figure 7b), wherein extracted cell culture medium solutions from PS and PS@Cu-MOF were serially diluted. For example, 100% refers to the original extraction medium, and 25% indicates a four times dilution from the original extraction medium. Following formation of the MEF monolayer, the culture medium was changed to the desired concentration medium containing the extract solution, and cultured. The obtained MTS results demonstrated the good cytocompatibilities of PS and PS@Cu-MOF, as the MEF viability was >93% in all cases, whereas the majority of MEFs died upon exposure to EtOH, thereby confirming the low cytotoxicity of the PS and PS@Cu-MOF extracts.

## 4. Conclusions

In this work, we successfully modified the surfaces of polysiloxanes (PSs) with copper-containing metal–organic frameworks (Cu-MOFs) via a hydrosilylation reaction. Modification was performed at room temperature without the requirement for any special equipment. The bactericidal activities of the Cu-MOF-embedded PS (i.e., PS@Cu-MOF) and the control PS were tested against three strains of bacteria, namely *E. coli*, *S. aureus*, and MRSA. Importantly, PS@Cu-MOF exhibited superior antibacterial properties toward the tested bacteria than the control PS, in addition to a low cytotoxicity toward mouse embryonic fibroblasts at a concentration of 100 μg⋅mL^−1^. In addition, Cu(II) ion release tests confirmed the excellent stability of PS@Cu-MOF in phosphate-buffered saline. Furthermore, the physical and thermal properties of PS@Cu-MOF, such as its phase transition, swelling ratio, and thermogravimetric profile, were comparable to those of the control PS. Moreover, its low cytotoxicity and high bactericidal activity indicate the potential of PS@Cu-MOF as a promising new candidate for medicinal applications, such as in implants, the treatment of skin disease, wound healing, and drug delivery. In summary, we attempted to efficiently improve the antibacterial activity of PS@Cu-MOF utilizing the synergy effect by blending with other additives, and we drove the development of another new bioactive PS. We believe that our findings could be useful for manufacturing a silicone-based implanting device with antibacterial activity in a straightforward and economically competitive way. However, further work, including in vivo experiments, is required to promote the future use of Cu-MOF-embedded PS as a new generation of powerful antibacterial agents and devices with wide-ranging applications.

## Figures and Tables

**Table 1:** Antibacterial activity of the MOF-embedded PS against various bacteria.

Test Items		Test Results ^a,b^		
		Early Conc. (cfu⋅mL^−1^)	After 24 h, Conc. (cfu⋅mL^−1^)	Reduction Rate of Bacteria (%)
*E. coli*	blank	3.4 × 10^5^	9.8 × 10^6^	-
	PS	3.4 × 10^5^	5.4 × 10^6^	44.8
	blank	1.2 × 10^5^	3.8 × 10^6^	-
	PS@Cu-MOF	1.2 × 10^5^	4.3 × 10^5^	88.6
*S. aureus*	blank	3.6 × 10^5^	9.1 × 10^6^	-
	PS	3.6 × 10^5^	6.2 × 10^6^	31.8
	blank	1.6 × 10^5^	2.2 × 10^6^	-
	PS@Cu-MOF	1.6 × 10^5^	2.7 × 10^5^	87.7
MRSA	blank	3.6 × 10^5^	9.0 × 10^6^	-
	PS	3.6 × 10^5^	7.2 × 10^6^	20.0
	blank	3.0 × 10^5^	9.8 × 10^5^	-
	PS@Cu-MOF	3.0 × 10^5^	1.8 × 10^5^	81.6

^a^ test method: KCL-FIR1003: 2018. ^b^ test environment: (37.0 ± 0.2) °C, Conc. 100 μg⋅mL^−1^.

## Data Availability

Data is available on the request from the corresponding author.

## Supplementary Materials

The following are available online at https://www.mdpi.com/2079-4991/11/3/719/s1, Figure S1: PXRD pattern of Cu-MOF, Figure S2: FT-IR spectrum of Cu-MOF, Figure S3: Crystal structure of Cu-MOF, Figure S4: FT-IR spectrum of PS and PS@Cu-MOF.
