# Supplementary material for: Robust Copper Metal–Organic Framework-Embedded Polysiloxanes for Biomedical Applications: Its Antibacterial Effects on MRSA and In Vitro Cytotoxicity
Source: Nanomaterials (Basel). 2021 Mar 12;11(3):719. doi: 10.3390/nano11030719 (PMC8000151; doi:10.3390/nano11030719)
Supplement: Supplementary file 1 [file nanomaterials-11-00719-s001.pdf]

nanomaterials

Supplementary Materials

Robust Copper Metal–Organic Framework-Embedded Polysiloxanes for Biomedical Applications: Its Antibacterial Effects on MRSA and In Vitro Cytotoxicity

Kihak Gwon [1], Youngmee Kim [2], Hyunjun Cho [3], Seonhwa Lee [1], So-Hyeon Yang [2], Sung-Jin Kim [2] and Do Nam Lee [1,*]

[1] Ingenium College of Liberal Arts (Chemistry), Kwangwoon University, Seoul 01897, Korea; khgwon@kw.ac.kr (K.G.); seonhwalee@kw.ac.kr (S.L.)
[2] Department of Chemistry and Nano Science, Institute of Nano-Bio Technology, Ewha Womans University, Seoul 03760, Korea, Seoul 03760, Korea; ymeekim@ewha.ac.kr (Y.K.); auung22@ewhain.net (S.-H.Y.); sjkim@ewha.ac.kr (S.-J.K.)
[3] Department of Chemistry, Dongguk University, Seoul 04620, Korea; vchol1212@dgu.ac.kr
* Correspondence: donamlee2@kw.ac.kr; Tel.: +82-2-940-5658

Citation: Gwon, K.; Kim, Y.; Cho, H.; Lee, S.; Yang, S.-H.; Kim, S.-J; Lee, D.N. Robust Copper Metal–Organic Framework-Embedded Polysiloxanes for Biomedical Applications: Its Antibacterial Effects on MRSA and In Vitro Cytotoxicity. *Nanomaterials* **2021**, *11*, 719. https://doi.org/10.3390/nano11030719

Academic Editor: Fernando Novio

Received: 14 February 2021
Accepted: 8 March 2021
Published: 12 March 2021

Publisher's Note: MDPI stays neutral with regard to jurisdictional claims in published maps and institutional affiliations.

Copyright: © 2021 by the authors. Licensee MDPI, Basel, Switzerland. This article is an open access article distributed under the terms and conditions of the Creative Commons Attribution (CC BY) license (http://creativecommons.org/licenses/by/4.0/).

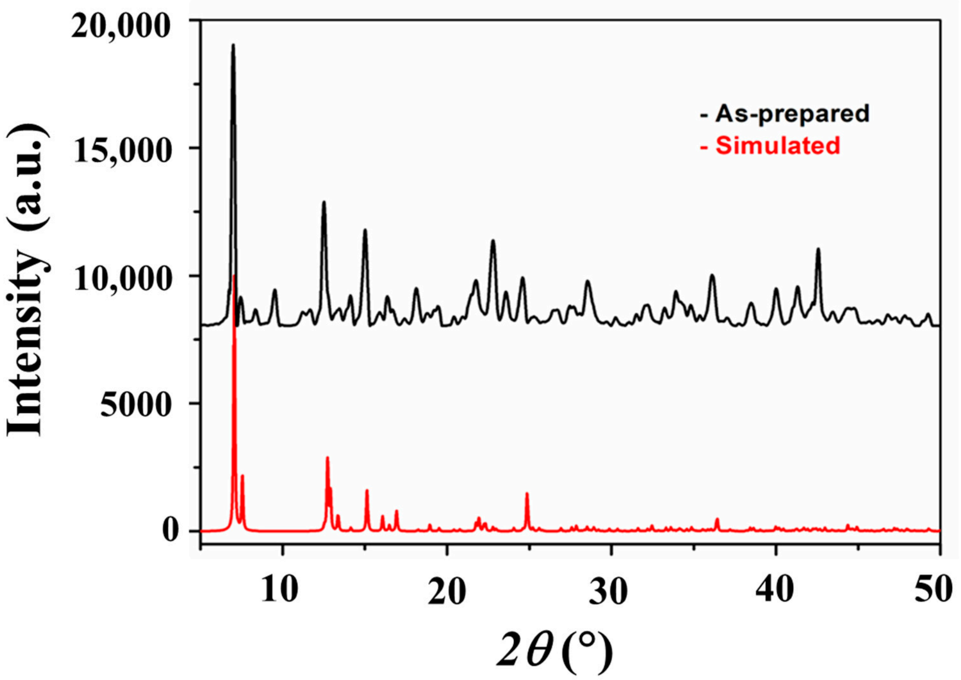

Figure S1. PXRD pattern of Cu-MOF.

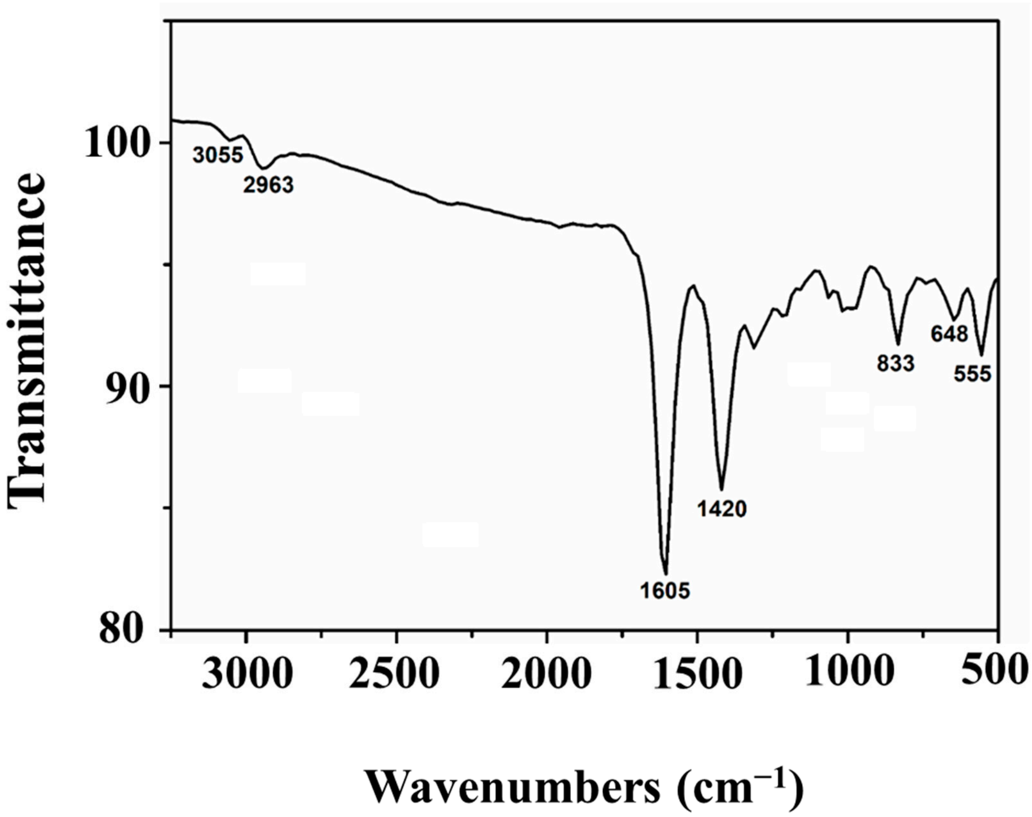

Figure S2. FT-IR spectrum of Cu-MOF.

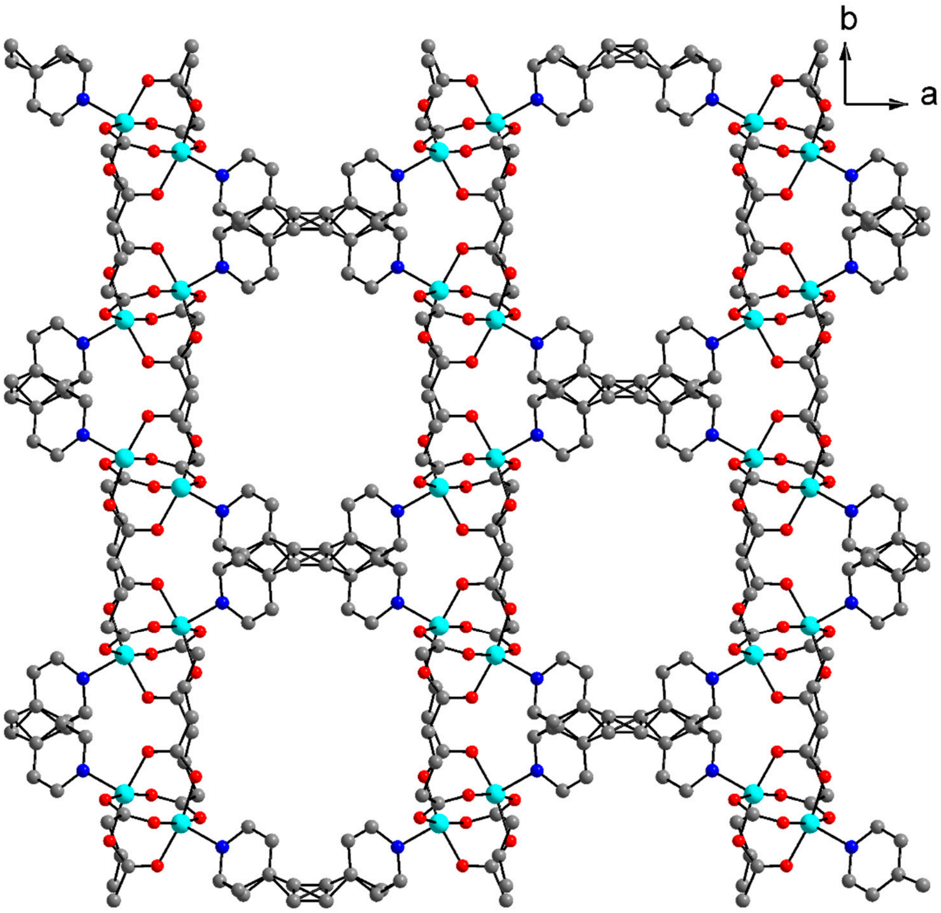

Figure S3. Crystal structure of Cu-MOF along the *c* axis. Water solvent molecules and hydrogen atoms are omitted for clarity. Color codes: green, copper; red, oxygen; blue, nitrogen; grey, carbon.

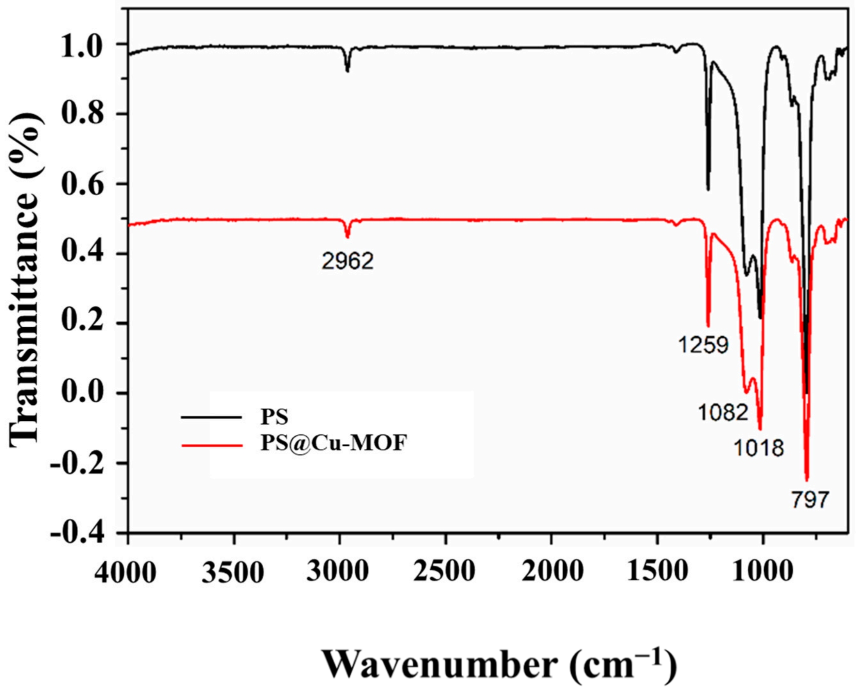

Figure S4. FT-IR spectra of PS (black) and PS@Cu-MOF (red).
